# The influence of athletes’ personality traits on competition performance: the multiple mediating effects of competitive anxiety, perfectionism, and stressors

**DOI:** 10.3389/fpsyg.2025.1724618

**Published:** 2026-01-13

**Authors:** Peng Dai, Cheng Chen, Mengyun Gu

**Affiliations:** 1 School of Physical Education, Huainan Normal University, Huainan, China; 2 Graduate School, Harbin Sport University, Harbin, China

**Keywords:** athletes, competition performance, competitive anxiety, perfectionism, personality traits, stressors

## Abstract

**Objective:**

To examine the impact of personality traits on athletes’ competition performance, verifying the dual-chain mediating roles of competitive anxiety, perfectionism, and stressors.

**Method:**

Using a questionnaire survey, personality traits, competitive anxiety, perfectionism, stressors, and competition performance were measured for 456 competitive aerobics athletes. The results were analyzed using mathematical statistics and structural equation modeling (SEM).

**Results:**

(1) Personality traits of openness, extraversion, conscientiousness, and agreeableness had a significant positive impact on athletes’ competitive anxiety, while neuroticism had a significant negative effect. (2) Traits of openness, extraversion, conscientiousness, and agreeableness also significantly positively influenced athletes’ perfectionism, whereas neuroticism significantly negatively impacted perfectionism. (3) Perfectionism had a significant positive effect on athletes’ competitive anxiety. (4) Competitive anxiety, perfectionism, and stressors played complete mediating roles between personality traits and athletes’ competition performance. Personality traits significantly impacted athletes’ competition performance not only through competitive anxiety and stressors as chain mediators but also through perfectionism and stressors as chain mediators. This study provides valuable theoretical insights and, more importantly, practical guidance for coaches and sports psychologists. The findings suggest that by assessing athletes’ personality traits, practitioners can develop tailored interventions to manage competitive anxiety, channel perfectionism adaptively, and help athletes reframe stressors as challenges rather than hindrances, ultimately optimizing their competition performance.

## 1 Introduction

The term “performance” originates from management science, referring to outcomes or benefits (Li et al., 2024). The 10th edition of the Oxford Advanced Learner’s English-Chinese Dictionary defines it as “execution, performance, fulfillment, achievement” (Hornby, 2023). The English term corresponding to performance is “performance,” defined in the Longman Dictionary as “the action of performing, or the action of performed,” which refers to the results of an ongoing or completed action (Li, 2019). High employee performance is valued and sought across all industries and sectors (Slåtten and Mehmetoglu, 2011). In the goal-oriented domain of competitive sports, coaches and athletes are particularly focused on achieving optimal competition performance. Competition performance is influenced by both “hard” factors, such as angiotensin-converting enzyme (ACE) genetic markers (Falahati and Arazi, 2019) and muscle fiber composition (Edman et al., 2019), and “soft” factors, such as personality traits (Noblet and Gifford, 2002).

Personality traits are relatively stable tendencies manifested in particular ways (Gebauer et al., 2020; Oklevik et al., 2020). Individuals with varying personalities exhibit distinct behavioral characteristics when pursuing personal goals (Clark et al., 2010). Studies indicate that personality traits are crucial for competition performance, serving as a “laboratory” to assess athletes’ responses under high pressure and intense emotions, thus creating unique conditions to study the role of individual differences in athletic performance (Zar et al., 2022). Perfectionism is considered a multidimensional personality trait characterized by an individual’s pursuit of flawlessness and excellence, often accompanied by critical self-evaluation or heightened sensitivity to mistakes (Hewitt and Flett, 1991). It is prevalent among athletes and significantly impacts their competition performance (Stoeber, 2011). Stoeber and colleagues found that striving for perfection is associated with improved goals and competition performance in young athletes, while perfectionistic concerns correlate with goal avoidance and decreased performance (Stoeber et al., 2009). Moreover, a significant relationship exists between perfectionism and competitive anxiety. For example, Hall and his research team (Hall et al., 1998) found that high school runners’ concerns over personal mistakes (an aspect of perfectionistic concerns) predict cognitive anxiety, whereas personal standards (a component of perfectionistic strivings) predict athletes’ confidence. Additionally, many athletes exhibit optimal performance in individual or team training but perform poorly in competitions. This discrepancy is attributed not only to personal technique and coaching skill but also to stressors that impact athletes’ mental states (Najafi et al., 2018). Relevant literature indicates that stressors are critical in influencing athletes’ competitive performance levels and are directly related to competition performance (Woodman and Hardy, 2001).

In the field of competitive sports, pursuing excellence and optimal athletic performance is a shared goal for coaches and athletes alike. Clarifying the influence of various personality traits on competition performance and applicable contexts aims to provide theoretical insights for coaching practices.

### 1.1 Theoretical foundation and research hypotheses

The Theory of Personality Trait (TPT), proposed by renowned American psychologist Allport in the 1940s, explores individual or group differences (Allport and Allport, 1921). This theory has shown stable patterns of cognition, emotion, and behavior across different situations over time, effectively reflecting individual behavioral tendencies (Primi et al., 2014; Hampson et al., 1986). Various models are used to study personality within psychology, among which Costa’s “Big Five” personality model—comprising the dimensions of Openness, Extraversion, Conscientiousness, Neuroticism, and Agreeableness—is one of the most influential (Costa, 1992). With the evolving competition environment, personality traits appear to be associated with athletes’ psychological characteristics (Acar, 2023). Research by Habib et al. indicates that openness, conscientiousness, and agreeableness in college athletes may serve as important predictors of competition performance (Habib et al., 2019).

Furthermore, a study by Zhang et al. (2019) confirms that female soccer players with conscientious and neurotic personality traits are more likely to become role model athletes. The psychological characteristics through which personality influences performance are multifaceted, but two critical mediators are competitive anxiety and perfectionism. Research indicates that personality is a key determinant of athletes’ responses in pressure-filled, evenly matched competitions (Robinson and Howe, 1987). For instance, a cross-sectional study by Kumar and Tankha (2022) on the relationship between Big Five personality traits and anxiety in 296 adults confirmed a significant impact of personality on anxiety, a finding likely extensible to the sports domain. Regarding perfectionism, research by Freire et al. (2022) suggests that it is essential for young athletes to develop their personality traits, as this contributes to forming a perfectionism-oriented goal approach. Stoeber (2014) identified socially prescribed perfectionism among college students as a unique form of perfectionism, highlighting a specific connection between social goals and personality traits. Previous studies have confirmed the influence of personality traits on competitive anxiety and perfectionism; however, the underlying mechanisms require further exploration.

**Figure 1:**
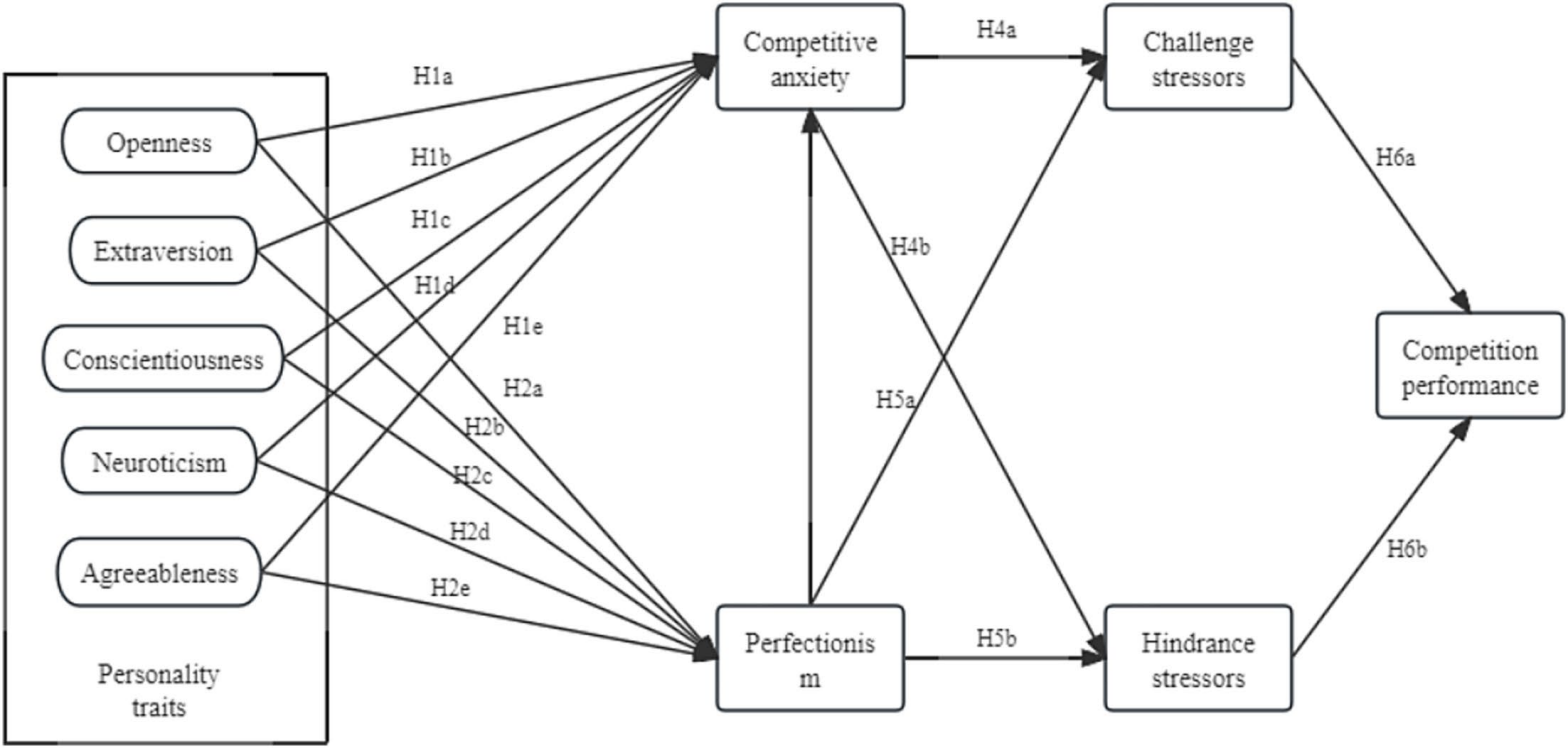
Theoretical model of the study.

Extensive literature shows that when competitive anxiety and perfectionism function as stressors, athletes who perceive a high likelihood of achieving their goals may experience positive effects (Hayes, 2009); conversely, those who anticipate failure may experience negative effects, thereby impacting their competition performance (Kouchaki and Desai, 2015). The Transactional Theory of Stress (TTS) posits that individuals’ responses to stressors are not determined by the stressor itself, but rather by their cognitive appraisal of it (Jiang and Wang, 2022). Selye (1978) research categorizes stress into “good” and “bad” forms. However, in competitive sports, not all stressors inhibit performance; some “positive” stressors may even enhance performance. Cavanaugh et al. (2000) further categorized stressors as challenge stressors and hindrance stressors based on their “positive” or “negative” nature. Rosen et al. (2020) found that when employees experience stable patterns of challenge stressors over time, these have a positive indirect impact on their performance and well-being. Research by Naseer et al. (2020) suggests that appraisals of hindrance stressors can reduce employee creativity, work engagement, and job performance (Sawhney and Michel, 2022), while increasing psychological stress and turnover intentions (Ma et al., 2021). In a meta-analysis of 183 independent samples, Podsakoff et al. (2007) found a positive correlation between challenge stressors and job performance, while hindrance stressors had the opposite relationship with job performance. Based on the Theory of Personality Trait and the Transactional Theory of Stress, this study constructs a theoretical model (Figure 1) with Big Five personality traits as independent variables, competitive anxiety, perfectionism, challenge stressors, and hindrance stressors as mediating variables, and competition performance as the dependent variable.

#### 1.1.1 Personality traits and competitive anxiety

Competitive anxiety arises from an imbalance between environmental demands and athletes’ coping abilities (Patel et al., 2010). In general, competitive anxiety is defined as an emotional state in which athletes experience worry and tension about their current situation (Martens and Schwenkmezger, 1979). Olefir (2018) study indicates that personality resources are systematically organized, forming a comprehensive factor at the empirical level, which helps to mitigate situational anxiety by partially acting on competitive stressors. Kemarat et al. (2022) found significant correlations between personality traits and competitive anxiety in both individual and team athletes, with neuroticism (*r* = −0.472, *p* < 0.001), extraversion (*r* = 0.184, *p* = 0.005), agreeableness (*r* = 0.147, *p* = 0.024), and conscientiousness (*r* = 0.202, *p* = 0.002) all significantly related to competitive anxiety. Other studies similarly confirm the influence of athletes’ personality traits on competitive anxiety (Pačesová, 2021; Santos-Rosa et al., 2022; Sfeir et al., 2022). Accordingly, this study proposes the following hypotheses:

*H1a*: Athletes’ openness positively influences competitive anxiety.

*H1b*: Athletes’ extraversion positively influences competitive anxiety.

*H1c*: Athletes’ conscientiousness positively influences competitive anxiety.

*H1d*: Athletes’ neuroticism negatively influences competitive anxiety.

*H1e*: Athletes’ agreeableness positively influences competitive anxiety.

#### 1.1.2 Personality traits and perfectionism

Perfectionism is a multidimensional personality trait encompassing a tendency to set excessively high performance standards and critically evaluate one’s behavior (Frost et al., 1990). Given the various conceptualizations of perfectionism (Terry-Short et al., 1995), factor analysis research has identified two higher-order dimensions: perfectionistic striving and perfectionistic concerns (Stoeber and Otto, 2006). Perfectionistic striving reflects a self-oriented drive to achieve high personal standards, while perfectionistic concerns involve worry over making mistakes, perception of discrepancies between standards and performance, and negative reactions to imperfection. These higher-order dimensions are measurable in the sports context (Stoeber and Madigan, 2016). Research has established links between specific dimensions of perfectionism and personality traits. For instance, theoretical and empirical reviews have noted associations between self-oriented perfectionism and traits such as neuroticism (Enns, 2002). Hogg et al. suggest that athletes with excessive perfectionism tend to set higher personal standards and demonstrate superior organizational skills, deriving satisfaction from their behaviors and experiencing less stress (Hogg, 1997). Accordingly, this study proposes the following hypotheses:

*H2a*: Athletes’ openness positively influences perfectionism.

*H2b*: Athletes’ extraversion positively influences perfectionism.

*H2c*: Athletes’ conscientiousness positively influences perfectionism.

*H2d*: Athletes’ neuroticism negatively influences perfectionism.

*H2e*: Athletes’ agreeableness positively influences perfectionism.

#### 1.1.3 Perfectionism and competitive anxiety

Previous research posits that perfectionism, as a unidimensional personality inclination, is indicative of maladaptive psychological adjustments and disorders, with individuals seeking support for depression and anxiety often displaying high levels of perfectionism (Stoeber, 2012). In sports, perfectionism is considered a common trait among champions (Gould et al., 2002). Perfectionism is also closely associated with competitive anxiety, achievement motivation, psychological fatigue, athletic performance, and other sports-related activities (Li and Zhang, 2007; Květon et al., 2021; Bao et al., 2010; Madigan et al., 2018). Hall et al.’s research shows that perfectionist athletes tend not only to complete coach-assigned training tasks but also to engage in additional training to enhance their performance, thereby reducing the negative effects of psychological stress on athletic performance (Hall et al., 1998). Furthermore, research distinguishing between adaptive and maladaptive aspects of perfectionism supports this nuanced view. Dale (2000), studying Swedish elite athletes, found that those with a profile of high effort but low concern over mistakes (akin to high strivings and low concerns) exhibited higher confidence and lower anxiety than those with a more maladaptive perfectionistic profile.

Accordingly, this study proposes the following hypothesis:

*H3*: Athletes’ perfectionism positively influences competitive anxiety.

#### 1.1.4 Competitive anxiety and stressors

In sports, athletes face numerous stressors, including pain, fear, low self-confidence, psychological demands, and pressure from coaches (Gould et al., 1993a, 1993b; Holt and Hogg, 2002). Anxiety is an individual’s response to stress (Speilberger, 1972), and athletes in competitive sports often exhibit behaviors related to stress. Research indicates that self-advancement and the pursuit of excellence are ideals and goals for athletes (Bucciol and Castagnetti, 2020). However, intense competition and high-stakes sports can lead to competitive anxiety, causing stress and decreased focus on the field (Sheard and Golby, 2006; Cole and Kvavilashvili, 2021). Liu et al., in their study on anxiety, mental stress, and burnout among Chinese college soccer players, found a significant positive correlation between athlete anxiety and mental stress (*r* = 0.126, *p* < 0.001) (Liu et al., 2022). Da Silva et al. (2021) suggest that mental training can regulate anxiety levels in Olympic shooters, helping them manage stress and achieve more consistent performance. Accordingly, this study proposes the following hypotheses:

*H4a*: Athletes’ competitive anxiety positively influences challenge-related stressors.

*H4b*: Athletes’ competitive anxiety negatively influences hindrance-related stressors.

#### 1.1.5 Perfectionism and stressors

According to Webster et al., challenge-related stressors provide individuals with opportunities to meet their basic needs, whereas hindrance-related stressors hinder individuals’ ability to meet these needs (Webster et al., 2010). In a similar context, personal needs can be viewed as either challenges or hindrances, depending on their nature. Challenge-related stressors (e.g., performance expectations, perfectionism) may fulfill an individual’s need for competence and achievement.

Self-oriented perfectionism in athletes subjects them to both physical and psychological pressures over time. These pressures include concerns over performance, fear of failure and dissatisfaction following losses, conflicts with coaches, partners, or family, and even the costs associated with training and physical demands—all of which significantly influence the sources of stress athletes experience (Cohn, 1990; Gould et al., 1993a, 1993b; Puente-Díaz and Anshel, 2005; Scanlan et al., 1991). Dunn et al. (2020), in monitoring athletes 24 h before a major competition, found that minor adaptive traits of perfectionism are related to the stressor assessment metrics of young soccer players. Accordingly, this study proposes the following hypotheses:

*H5a*: Athletes’ perfectionism positively influences challenge-related stressors.

*H5b*: Athletes’ perfectionism negatively influences hindrance-related stressors.

#### 1.1.6 Stressors and performance

Cavanaugh et al. (2000) categorize stressors into two types: challenge-related stressors and hindrance-related stressors. Challenge-related stressors include those associated with positive outcomes, such as workload, time urgency, job responsibility, and job complexity, which are common in work settings (O'Brien and Beehr, 2019; Abbas and Raja, 2019). Challenge-related stressors are perceived as surmountable work-related demands, often aiding individuals in achieving success. They are associated with potential personal gains and growth (Duan et al., 2011; Alam and Singh, 2021). Sun et al., through a qualitative comparative analysis of 240 valid paired samples from employees in three Chinese companies, found a direct positive impact of challenge-related stressors on employee creativity (Sun et al., 2019). Hindrance-related stressors, on the other hand, limit personal development and goal achievement (Cavanaugh et al., 2000), such as role ambiguity, lack of performance guidance, organizational politics, and insufficient resources (Podsakoff et al., 2007). Hindrance-related stressors are regarded as negative work experiences, eroding employees’ sense of control and autonomy, diverting their focus from work, reducing enthusiasm, and ultimately harming overall job satisfaction and performance (LePine et al., 2016; Zhang et al., 2018). Zhu et al. (2017) in examining the effects of hindrance-related stressors on academic performance among southern Chinese college students, found a negative correlation between hindrance stress and academic performance. Accordingly, this study proposes the following hypotheses:

*H6a*: Challenge-related stressors positively influence athletes' performance.

*H6b*: Hindrance-related stressors negatively influence athletes' performance.

## 2 Research subjects and methods

### 2.1 Research subjects

A significant body of literature indicates a moral hazard of free-riding in team sports (Albanese and Van Fleet, 1985), as aerobics athletes tend to have a stronger goal orientation towards performance and more direct evaluations. Considering this, 496 aerobics athletes from 17 provinces (municipalities and autonomous regions) including Anhui, Jiangsu, Zhejiang, Shanghai, and Sichuan were selected as the research sample. A total of 456 valid questionnaires were collected, yielding an effective response rate of 91.9%. Of the respondents, 258 were male athletes (56.6%) and 198 were female athletes (43.4%). The average age was 21.08 ± 1.54 years, and the average training experience was 5.41 ± 1.61 years. Regarding specialization, 194 participated in individual events (42.5%) and 262 in group events (57.5%). In terms of athletic level, there were 200 athletes at the national second level (43.9%), 123 at the national first level (27.0%), 87 national athletes (19.1%), and 46 without ranking (10.1%). Regarding athletic talent, 97 athletes were rated as excellent (21.3%), 123 as good (27.0%), 167 as average (36.6%), 47 as below average (10.3%), and 22 as poor (4.8%). Weekly training frequency was as follows: once a week (14 athletes, 3.1%), twice a week (40, 8.8%), three times a week (47, 10.3%), four times a week (84, 18.4%), five times a week (105, 23.0%), six times a week (95, 20.8%), and seven times a week (71, 15.6%). Regarding training attitudes, 311 athletes (68.2%) considered training essential, 103 (22.6%) saw it as routine, 29 (6.4%) as optional, and 13 (2.9%) as obligatory. For accompanying personnel besides the head coach, 28 athletes (6.1%) had none, 56 (12.3%) had one, 125 (27.4%) had two, 143 (31.4%) had three, and 104 (22.8%) had four or more.

### 2.2 Measures

All constructs were measured using established scales adapted to the sports context. Items were rated on a 7-point Likert-type scale (1 = Strongly Disagree, 7 = Strongly Agree).

Personality Traits. We used the Chinese version of the NEO Five-Factor Inventory (Zhang and Zhou, 2006), which has been validated in Chinese populations. The scale includes 54 items measuring five dimensions: Openness, Extraversion, Conscientiousness, Neuroticism, and Agreeableness. A sample item is “I enjoy trying new and foreign foods.” The scale demonstrated acceptable reliability in this study (*α* = 0.72).

Competitive Anxiety. This was measured using a Chinese adaptation of the Competitive State Anxiety Inventory-2 (CSAI-2) (Martens, 1990) that has been widely used in sport psychology research with Chinese athletes. The 27-item scale assesses three components: Cognitive State Anxiety (e.g., “I am concerned about performing poorly”), Somatic State Anxiety, and State Self-Confidence. Its Cronbach’s *α* was 0.80 in this study.

Perfectionism. We employed the Sport-Specific Version of the Multidimensional Perfectionism Scale (MPS-S), which was translated and validated for Chinese athletes by Lian (2007). This 37-item scale comprises five dimensions: Personal Standards, Rumination, Concern over Mistakes, Perceived Parental Pressure, and Perceived Coach Pressure. It captures aspects of both perfectionistic strivings (e.g., Personal Standards) and concerns (e.g., Concern over Mistakes). The overall scale reliability in this study was α = 0.85.

Stressors. We adapted the 11-item scale from Cavanaugh et al. (2000) to the sports context. The original scale has demonstrated good psychometric properties in organizational settings, and its Chinese translation has been used in prior research. For this study, items were reworded to refer explicitly to the athletic training and competition environment (e.g., “The high level of responsibility in competition”). The scale measures Challenge Stressors (6 items) and Hindrance Stressors (5 items). Both subscales showed excellent reliability in this study (*α* = 0.92 each).

Competition Performance. A 10-item self-report scale was developed to assess athletes’ perceived performance effectiveness. Following Campbell’s (1990) critical incident technique, we first interviewed expert coaches and high-level athletes to identify key performance indicators in competitive aerobics (e.g., technical execution, artistic expression, consistency under pressure). Based on these indicators, items were crafted (e.g., “I consistently achieve the technical difficulty goals set by my coach,” “My artistic expression in the routine meets competition standards,” “I perform reliably under competitive pressure”). Participants rated their agreement with each statement regarding their performance during the most recent competition season on a 7-point scale (1 = Strongly Disagree, 7 = Strongly Agree). The scale demonstrated excellent reliability (*α* = 0.93).

### 2.3 Research model

Guided by the Theory of Personality Trait and the Transactional Theory of Stress, this study proposes the dual-chain mediation model illustrated in Figure 1. The model posits that athletes’ Big Five personality traits influence competition performance through two parallel psychological pathways: one involving competitive anxiety and the other involving perfectionism. These psychological states, in turn, are theorized to shape the appraisal of situational demands as either challenge stressors or hindrance stressors, which finally affect performance.

### 2.4 Data analysis strategy

The data analysis followed a sequential process using SPSS 23.0 and Amos 21.0. First, preliminary analyses including descriptive statistics and scale reliability (Cronbach’s α) were conducted. Second, the measurement model was evaluated using Confirmatory Factor Analysis (CFA) to assess convergent and discriminant validity. Third, Structural Equation Modeling (SEM) was employed to test the hypothesized relationships in the structural model and to evaluate overall model fit. SEM was chosen as the primary analytical technique because it allows for the simultaneous estimation of multiple interrelated dependence relationships among latent variables while accounting for measurement error. Finally, to test the significance of the proposed indirect (mediation) effects, we used the nonparametric bias-corrected bootstrap method with 2000 resamples. This method is preferred as it does not assume a normal distribution of the indirect effect and provides robust confidence intervals.

## 3 Analysis results

### 3.1 Preliminary analysis: evaluation of the measurement model

#### 3.1.1 Reliability testing

This study applied confirmatory factor analysis to evaluate the measurement model, focusing on composite reliability (CR values) and internal consistency (Cronbach’s α values). As shown in Table 1, the CR values for the constructs of personality traits, competitive anxiety, perfectionism, challenge-related stressors, hindrance-related stressors, and performance are 0.72, 0.81, 0.85, 0.92, 0.92, and 0.93, respectively, all above the recommended threshold of 0.70. Similarly, the Cronbach’s α values for these constructs are 0.72, 0.80, 0.85, 0.92, 0.92, and 0.93, all exceeding the 0.70 benchmark, indicating that the questionnaires and sample data in this study demonstrate high reliability.

**Table 1:** Summary of confirmatory factor analysis results.

Latent variables	Observed variables	Significance parameters				Std.	SMC	CR	AVE	Cronbach’s *α*
		Unstd.	S.E.	Z	*p*					
PT	O	1.00				0.66	0.43	0.72	0.34	0.72
	E	0.70	0.09	7.90	***	0.48	0.23			
	C	0.86	0.10	8.87	***	0.56	0.32			
	N	0.95	0.10	9.47	***	0.63	0.39			
	A	0.88	0.10	9.10	***	0.59	0.34			
CA	CSA	1.00				0.72	0.51	0.81	0.58	0.80
	SSA	1.01	0.08	13.27	***	0.74	0.54			
	SSC	1.12	0.08	13.32	***	0.83	0.69			
P	PS	1.00				0.75	0.57	0.85	0.53	0.85
	RTM	1.00	0.07	14.57	***	0.73	0.53			
	CM	0.94	0.07	13.94	***	0.70	0.49			
	PPS	0.98	0.07	14.31	***	0.72	0.52			
	PCS	0.98	0.07	14.55	***	0.73	0.53			
CS	CS1	1.00				0.82	0.66	0.92	0.66	0.92
	CS2	0.97	0.05	19.48	***	0.80	0.64			
	CS3	1.01	0.05	20.08	***	0.82	0.67			
	CS4	0.99	0.05	19.99	***	0.82	0.67			
	CS5	0.97	0.05	19.34	***	0.80	0.64			
	CS6	1.00	0.05	19.53	***	0.80	0.65			
HS	HS1	1.00				0.82	0.68	0.92	0.69	0.92
	HS2	1.03	0.05	21.27	***	0.85	0.72			
	HS3	1.01	0.05	21.09	***	0.84	0.71			
	HS4	0.95	0.05	20.48	***	0.82	0.68			
	HS5	0.95	0.05	19.84	***	0.81	0.65			
CP	CP1	1.00				0.76	0.58	0.93	0.57	0.93
	CP2	0.97	0.06	16.63	***	0.75	0.57			
	CP3	0.97	0.06	17.07	***	0.77	0.59			
	CP4	0.96	0.06	16.38	***	0.74	0.55			
	CP5	0.95	0.06	16.33	***	0.74	0.55			
	CP6	1.05	0.06	17.11	***	0.77	0.60			
	CP7	0.99	0.06	16.94	***	0.77	0.59			
	CP8	0.91	0.06	15.81	***	0.72	0.52			
	CP9	1.00	0.06	16.21	***	0.74	0.54			
	CP10	0.99	0.06	16.69	***	0.76	0.57			

PT represents Personality Traits; CA stands for Competitive Anxiety; P denotes Perfectionism; CS indicates Challenge-related Stressors; HS refers to Hindrance-related Stressors; CP signifies Performance; *** denotes *p* < 0.001.

#### 3.1.2 Validity testing

Validity testing in this study primarily assessed convergent and discriminant validity. Average variance extracted (AVE) was employed to evaluate the convergent and discriminant validity of each latent variable. Convergent validity reflects the degree of correlation among indicators within a measurement tool (Hair et al., 2006). An AVE value exceeding 0.5 suggests good convergent validity for a latent variable. As shown in Table 1, except for personality traits, the AVE values for other constructs range from 0.53 to 0.69, all above Fornell and Larcker (1981) minimum standard of 0.5. The lower AVE for personality traits may be due to the high number of observed variables. This indicates that the constructs and model exhibit satisfactory convergent validity.

Discriminant validity refers to the distinctiveness of different variables in terms of their characteristics (Green and Carroll, 1978). When the square root of AVE exceeds the correlation coefficients between paired constructs, good discriminant validity is indicated. As shown in Table 2, except for personality traits, the square roots of AVE for the remaining variables are all greater than their correlations with other variables, confirming adequate discriminant validity among variables. Overall, the measurement model in this study demonstrates good validity, supporting its use as a tool for relevant research.

**Table 2:** Discriminant validity analysis.

	AVE	PT	CA	P	CS	HS	CP
PT	0.343	***0.586***					
CA	0.583	0.636***	***0.764***				
P	0.528	0.598***	0.511***	***0.727***			
CS	0.655	0.521***	0.444***	0.471***	***0.809***		
HS	0.686	−0.492***	−0.493***	−0.427***	−0.348***	***0.828***	
CP	0.566	0.467***	0.429***	0.377***	0.382***	−0.311***	***0.752***

*** indicates *p* < 0.001; bold italics represent the square root of AVE.

### 3.2 Structural model and hypothesis testing

#### 3.2.1 Structural model fit test

**Figure 2:**
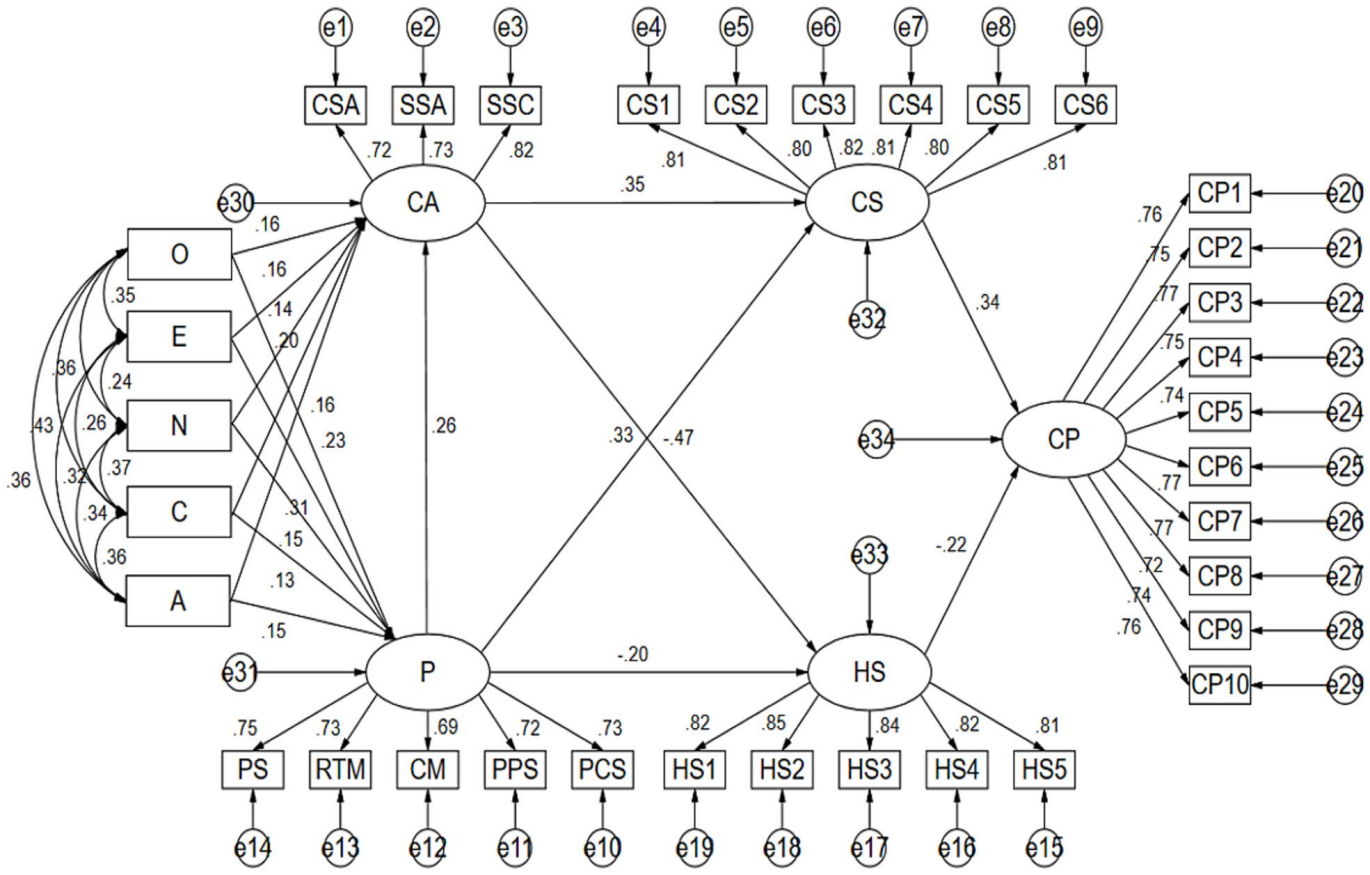
Structural equation model diagram.

The goodness-of-fit test for the structural model among the latent variables yielded the following results: χ^2^ = 583.974, df = 505, χ^2^/df = 1.156 < 3, indicating an ideal fit; RMSEA = 0.019, which is less than 0.08, also indicating an ideal fit; GFI = 0.933, AGFI = 0.921, CFI = 0.991, NFI = 0.935, RFI = 0.928, IFI = 0.991, TLI = 0.990, all exceeding the 0.9 fit standard. Overall, the structural model demonstrates a good fit (Figure 2).

#### 3.2.2 Hypothesis testing

According to the results shown in Table 3, all proposed hypotheses were validated through significance testing. Additionally, most dimensions and their corresponding items were positively correlated, with a reverse coding applied for neuroticism, so positive values indicate negative effects. Specifically: Openness to experience had a significant positive effect on competitive anxiety (*β* = 0.216, *p* = 0.001), confirming Hypothesis H1a, which states that openness is positively correlated with competitive anxiety; extraversion also had a significant positive effect on competitive anxiety (*β* = 0.236, *p* < 0.01), confirming Hypothesis H1b, meaning that extraversion is positively correlated with competitive anxiety; conscientiousness had a significant positive effect on competitive anxiety (*β* = 0.232, *p* < 0.001), validating Hypothesis H1c, indicating that conscientiousness is positively correlated with competitive anxiety; neuroticism, however, showed a significant negative effect on competitive anxiety (*β* = 0.175, *p* = 0.002), supporting Hypothesis H1d, which suggests that neuroticism is negatively correlated with competitive anxiety; agreeableness had a significant positive effect on competitive anxiety (*β* = 0.202, *p* < 0.001), confirming Hypothesis H1e, indicating that agreeableness is positively correlated with competitive anxiety; openness to experience also had a significant positive effect on perfectionism (*β* = 0.232, *p* < 0.001), validating Hypothesis H2a, which posits a positive correlation between openness and perfectionism; extraversion had a significant positive effect on perfectionism (*β* = 0.305, *p* < 0.001), confirming Hypothesis H2b, suggesting that extraversion is positively correlated with perfectionism; conscientiousness was also positively correlated with perfectionism (*β* = 0.134, *p* = 0.004), supporting Hypothesis H2c; neuroticism had a significant negative effect on perfectionism (*β* = 0.149, *p* = 0.001), validating Hypothesis H2d, which suggests a negative correlation between neuroticism and perfectionism; agreeableness positively influenced perfectionism (*β* = 0.150, *p* = 0.001), confirming Hypothesis H1e, which implies a positive correlation between agreeableness and perfectionism; perfectionism was positively correlated with competitive anxiety (*β* = 0.264, *p* < 0.001), confirming Hypothesis H3; competitive anxiety had a significant positive effect on challenge-related stressors (*β* = 0.349, *p* < 0.001), validating Hypothesis H4a, indicating a positive correlation between competitive anxiety and challenge-related stressors; competitive anxiety had a significant negative effect on hindrance-related stressors (*β* = −0.458, *p* < 0.001), confirming Hypothesis H4b, suggesting a negative correlation between competitive anxiety and hindrance-related stressors; perfectionism was positively correlated with challenge-related stressors (*β* = 0.353, *p* < 0.001), validating Hypothesis H5a; perfectionism had a significant negative effect on hindrance-related stressors (*β* = −0.233, *p* = 0.002), confirming Hypothesis H5b; challenge-related stressors positively impacted athletic performance (*β* = 0.338, *p* < 0.001), confirming Hypothesis H6a, suggesting a positive correlation between challenge-related stressors and athletic performance; hindrance-related stressors negatively affected athletic performance (*β* = −0.222, *p* < 0.001), confirming Hypothesis H6b, indicating a negative correlation between hindrance-related stressors and athletic performance.

**Table 3:** Path analysis and research hypothesis results.

H₀	Path	SP	t	*p*	Conclusion
H1a	Openness → competitive anxiety	0.216	3.263	0.001	Support
H1b	Extraversion → competitive anxiety	0.236	3.420	<0.001	Support
H1c	Conscientiousness → competitive anxiety	0.232	4.267	<0.001	Support
H1d	Neuroticism → competitive anxiety	0.175	3.063	0.002	Support
H1e	Agreeableness → competitive anxiety	0.202	3.618	<0.001	Support
H2a	Openness → perfectionism	0.232	4.820	<0.001	Support
H2b	Extraversion → perfectionism	0.305	6.687	<0.001	Support
H2c	Conscientiousness → perfectionism	0.134	2.847	0.004	Support
H2d	Neuroticism → perfectionism	0.149	3.268	0.001	Support
H2e	Agreeableness → perfectionism	0.150	3.257	0.001	Support
H3	Perfectionism → competitive anxiety	0.264	4.258	<0.001	Support
H4a	Competitive anxiety → challenge stressors	0.349	5.203	<0.001	Support
H4b	Competitive anxiety → hindrance stressors	−0.458	−6.677	<0.001	Support
H5a	Perfectionism → challenge stressors	0.353	4.981	<0.001	Support
H5b	Perfectionism → hindrance stressors	−0.233	−3.059	0.002	Support
H6a	Challenge stressors → competition performance	0.338	6.447	<0.001	Support
H6b	Hindrance stressors → competition performance	−0.222	−4.375	<0.001	Support

#### 3.2.3 Mediation effect testing

The model adopts a double-chain mediation model, with the nonparametric percentile bootstrap method (bias-corrected) as the most appropriate testing method. Using 2000 bootstrap samples to calculate the 95% confidence interval (CI), the results in Table 4 show an estimated value of 0.018, standard error of 0.010, and Z value of 1.800 for the openness → competitive anxiety → challenge-related stressors → athletic performance path. At the 95% confidence level, the bias-corrected confidence interval lower bound was 0.003 and upper bound was 0.044. Since zero was not included and the *p*-value was less than 0.05, this mediation effect was established. Similarly, all other mediation effects were found significant. Therefore, competitive anxiety, perfectionism, and stressors served as mediators, indicating that this model constitutes full mediation.

**Table 4:** Mediation effect analysis results.

Path	PE (point estimate)	SE	Z	Bias-corrected 95% CI		*P*
				Lower	Upper	
Openness → competitive anxiety → challenge stressors → competition performance	0.018	0.010	1.800	0.003	0.044	0.008
Extraversion → competitive anxiety → challenge stressors → competition performance	0.019	0.009	2.111	0.006	0.045	0.006
Neuroticism → competitive anxiety → challenge stressors → competition performance	0.016	0.009	1.778	0.003	0.041	0.013
Conscientiousness → competitive anxiety → challenge stressors → competition performance	0.023	0.011	2.091	0.007	0.053	0.001
Agreeableness → competitive anxiety → challenge stressors → competition performance	0.019	0.009	2.111	0.006	0.044	0.003
Openness → competitive anxiety → hindrance stressors → competition performance	0.016	0.009	1.778	0.004	0.040	0.007
Extraversion → competitive anxiety → hindrance stressors → competition performance	0.017	0.009	1.889	0.005	0.041	0.006
Neuroticism → competitive anxiety → hindrance stressors → competition performance	0.014	0.008	1.750	0.003	0.037	0.010
Conscientiousness → competitive anxiety → hindrance stressors → competition performance	0.020	0.010	2.000	0.007	0.047	0.001
Agreeableness → competitive anxiety → hindrance stressors → competition performance	0.017	0.009	1.889	0.005	0.045	0.003
Openness → perfectionism → challenge stressors → competition performance	0.025	0.012	2.083	0.009	0.058	0.002
Extraversion → perfectionism → challenge stressors → competition performance	0.035	0.012	2.917	0.015	0.065	0.002
Neuroticism → perfectionism → challenge stressors → competition performance	0.016	0.009	1.778	0.004	0.041	0.004
Conscientiousness → perfectionism → challenge stressors →competition performance	0.015	0.010	1.500	0.002	0.040	0.015
Agreeableness → perfectionism → challenge stressors → competition performance	0.016	0.008	2.000	0.005	0.039	0.004
Openness → perfectionism → hindrance stressors → competition performance	0.010	0.006	1.667	0.002	0.029	0.013
Extraversion → Perfectionism → Hindrance stressors → competition performance	0.014	0.007	2.000	0.003	0.034	0.012
Neuroticism → perfectionism → hindrance stressors → competition performance	0.006	0.004	1.500	0.001	0.020	0.013
Conscientiousness → perfectionism → hindrance stressors → competition performance	0.006	0.004	1.500	0.001	0.021	0.020
Agreeableness → perfectionism → challenge stressors →competition performance	0.007	0.005	1.400	0.001	0.022	0.016

## 4 Discussion

### 4.1 Athlete personality traits directly explain competitive anxiety

This study shows that openness, extraversion, conscientiousness, and agreeableness significantly and positively influence athletes’ competitive anxiety, while neuroticism has a significant negative impact. These findings provide strong support for the Theory of Personality Trait (TPT), demonstrating that stable individual differences systematically predict emotional responses in competitive settings. These findings align with previous studies, suggesting that personality traits affect athletes’ competitive anxiety and that the impact varies across different personality dimensions (Petito et al., 2016). Specifically, conscientiousness has the highest path coefficient for competitive anxiety at 0.20, slightly higher than openness, extraversion, and agreeableness, each with a coefficient of 0.16. Conscientious athletes are driven to control their anxiety and focus on their goals, thus displaying a high sense of responsibility and striving to succeed optimally in sports (Eysenck and Eysenck, 2013). Conversely, neuroticism is characterized by a tendency to experience negative emotions, such as anger, anxiety, or depression, which may relate to increased anxiety in athletes under high psychological stress. According to personality trait theory (Mitchell and Kumari, 2016; Kim et al., 2019), neuroticism is associated with low tolerance for stress or stimuli. Athletes with high neuroticism scores may be emotionally reactive and easily affected by stress. Additionally, athletes’ anxiety can be influenced by other determinants, depending on the level of exposure to competitive experiences. Athletes appear to have specific needs, such as intensive training demands and psychological pressures, which are associated with elevated anxiety levels (Safari et al., 2019).

### 4.2 Athlete personality traits directly explain perfectionism

This study reveals that athletes’ personality traits directly influence perfectionism. Openness, extraversion, conscientiousness, and agreeableness all have a positive impact on perfectionism among athletes. This reinforces the TPT perspective, showing that core personality dimensions shape the development of secondary, domain-specific traits like perfectionism in sports. Extraversion has the closest association with perfectionism, with a standardized path coefficient of 0.31. This is likely because extroverted athletes actively engage in social activities, gaining additional energy through their interactions with others. Previous research indicates that perfectionistic athletes set higher personal standards (McCrae and Costa, 2004) and often require additional training to enhance their competitive performance (Stoeber and Otto, 2006). This study also indicates a negative effect of neuroticism on athletes’ perfectionism. Neurotic athletes tend to be timid, prone to panic and anxiety, and are often unable to cope with daily training pressures, which in turn affects their ability to achieve perfection in competition (Gould et al., 2002). These findings are supported by previous studies, which suggest that athletes who strive for perfection derive satisfaction from their actions and experience lower stress (McCrae and Costa, 2004). In contrast, athletes pursuing perfection due to high external expectations may face psychological pressure, which can negatively impact their performance (Hall et al., 1998; Hill et al., 2015).

### 4.3 Athlete perfectionism positively influences competitive anxiety

This study demonstrates that perfectionism in athletes significantly and positively influences competitive anxiety, with a standardized path coefficient of 0.26, consistent with previous findings (Jones, 1995). This relationship highlights how a maladaptive cognitive-behavioral style (perfectionism) can exacerbate situational anxiety, a connection central to understanding the psychological pathways outlined in our model. The reason for this relationship is that athletes competing at high levels often consider themselves perfectionists and view perfectionism as a key source of their success (Madigan et al., 2017). Furthermore, sports often require peak performance under significant pressure. Additionally, athletes’ experiences, particularly during competitions, along with the close scrutiny of coaches, teammates, and parents, may also contribute to heightened competitive anxiety (Folkman, 1984). Therefore, athletes with perfectionistic traits are more prone to psychological disorders, such as anxiety.

### 4.4 Chain mediation effects of competitive anxiety, perfectionism, and stressors

This study indicates that competitive anxiety, perfectionism, and stressors act as a chain mediator in the relationship between personality traits and athletes’ performance. These chain mediation effects are best understood through the lens of the Transactional Theory of Stress (TTS). Competitive anxiety and stressors serve as complete mediators between personality traits and athletic performance. According to the stress interaction theory, individuals who evaluate stressors as challenging are less likely to be disrupted by anxiety and tend to focus more on problem-solving, while those who assess stressors as hindrances are more prone to anxiety (Abbas and Raja, 2019). Specifically, as a stressor, athletes’ competitive anxiety arising from challenge-related stressors increases competition difficulty, mental exhaustion, and anxiety. However, it also fosters a sense of honor, encourages self-motivation, and creates a strong drive to enhance performance, providing athletes with growth opportunities and fostering exceptional performance. Consequently, athletes are more willing to invest energy to confront challenge-related stressors, whereby competitive anxiety positively influences challenge-related stressors and subsequently impacts performance. Conversely, when athletes face hindrance-related stressors that not only drain energy but also hinder outstanding performance, they may avoid or withdraw energy investment, allowing anxiety to dominate. Their perceived situation becomes more threatening than the actual scenario, whereby competitive anxiety negatively affects hindrance-related stressors, thereby impacting performance.

Perfectionism and stressors act as complete mediators between personality traits and athletes’ performance. Based on the stress interaction theory, when stressors are assessed as potentially challenging, individuals tend to adopt positive coping strategies, such as doubling efforts to meet situational demands. However, when individuals encounter crises that are difficult to manage, they are more likely to form hindrance appraisals, including reduced self-efficacy and avoidance behaviors (Dawson et al., 2016). Specifically, as an alternative source of stress, athletes’ perfectionism causes them to face continuous competition pressure, leading to the depletion of some internal resources, such as psychological and emotional resilience. At this point, self-regulatory mechanisms can help them seek external resources to change their competitive state and better achieve their performance goals, whereby perfectionism positively influences challenge-related stressors and subsequently affects performance. In contrast, the high standards and strict requirements that accompany individual perfectionism place athletes under sustained competition pressure, depriving them of time originally reserved for relaxation. This state leads them to perceive the situation as unfavorable, triggering negative emotions and exacerbating performance burnout, whereby perfectionism negatively affects hindrance-related stressors and subsequently impacts performance.

### 4.5 Limitations and future directions

Despite its contributions, this study has several limitations that should be acknowledged. First, the cross-sectional design precludes causal inferences. While our model is theoretically grounded, longitudinal or experimental studies are needed to confirm the directionality of the proposed pathways. Second, the reliance on self-reported data for all constructs may introduce common method bias, although we employed procedural remedies (e.g., ensuring anonymity) and the model fit was good. Future research could benefit from multi-method assessments, such as integrating coach ratings for performance or physiological measures for anxiety.

Third, while we used established or carefully adapted scales, the cross-cultural validity of some constructs (particularly the challenge-hindrance stressor framework) within Chinese athletic populations warrants further psychometric investigation. Fourth, our study treated perfectionism as a global construct for model parsimony. Future work should disentangle the distinct roles of perfectionistic strivings and concerns, as they may have differential effects on anxiety, stress appraisal, and performance.

Fifth, although our sample included both individual and group event athletes, the sample size within each sub-category limited our ability to test for meaningful differences between them. Future studies with larger subgroup samples could explore whether the identified psychological pathways vary by event type. Finally, as the reviewer astutely noted, the rich dataset lends itself to person-centered analytic approaches (e.g., latent class or profile analysis) to identify subgroups of athletes with distinct combinations of personality traits and psychological profiles. Such exploratory analyses could yield nuanced insights beyond the variable-centered model tested here and represent a promising avenue for future research.

### 4.6 Practical implications for coaches

The findings of this study offer several actionable insights for coaches and sport psychologists working with aerobics athletes and similar populations.

First, the significant influence of personality traits on competitive anxiety and perfectionism suggests that personality assessment can be a valuable tool in an athlete’s psychological profile. Coaches can use this information to anticipate an athlete’s likely stress reactions and perfectionistic tendencies, allowing for more personalized communication and support strategies.

Second, the dual nature of perfectionism implies that coaches should strive to foster adaptive perfectionistic strivings (e.g., setting high but realistic personal standards) while helping athletes mitigate maladaptive perfectionistic concerns (e.g., fear of mistakes, rumination). This can be achieved through cognitive-reframing techniques and process-oriented feedback.

Third, the chain mediation through stressors highlights the critical role of cognitive appraisal. Coaches can help athletes reinterpret potentially threatening competitive demands as challenges to be mastered rather than hindrances to be feared. Mental skills training focused on stress reappraisal and resource-building can be integrated into regular practice.

In summary, moving beyond a one-size-fits-all approach, practitioners can develop more effective, individualized interventions by considering the interplay between an athlete’s personality, their psychological states (anxiety, perfectionism), and how they perceive competitive pressures.

## 5 Conclusion

First, personality traits significantly impact athletes’ competitive anxiety, with openness, extraversion, conscientiousness, and agreeableness having a positive effect on competitive anxiety, whereas neuroticism has a significant negative impact on competitive anxiety. Second, personality traits significantly influence athletes’ perfectionism, with openness, extraversion, conscientiousness, and agreeableness having a positive effect on perfectionism, while neuroticism has a negative impact on perfectionism. Third, perfectionism has a significant positive impact on athletes’ competitive anxiety. Fourth, competitive anxiety and stressors serve as chain mediators between personality traits and athletic performance. Specifically, competitive anxiety and challenge-related stressors act as full mediators between personality traits and athletic performance, as do competitive anxiety and hindrance-related stressors. Fifth, perfectionism and stressors serve as chain mediators between personality traits and athletic performance. Specifically, perfectionism and challenge-related stressors act as full mediators between personality traits and athletic performance, as do perfectionism and hindrance-related stressors.

## Data Availability

The raw data supporting the conclusions of this article will be made available by the authors, without undue reservation.
